# Machine Learning-Assisted Discovery of Empirical Rule for Martensite Transition Temperature of Shape Memory Alloys

**DOI:** 10.3390/ma18102226

**Published:** 2025-05-12

**Authors:** Hao-Xuan Liu, Hai-Le Yan, Nan Jia, Bo Yang, Zongbin Li, Xiang Zhao, Liang Zuo

**Affiliations:** Key Laboratory for Anisotropy and Texture of Materials (Ministry of Education), School of Material Science and Engineering, Northeastern University, Shenyang 110819, China; liuhx_neu@yeah.net (H.-X.L.); jian@atm.neu.edu.cn (N.J.); yangb@atm.neu.edu.cn (B.Y.); lizb@atm.neu.edu.cn (Z.L.); zhaox@mail.neu.edu.cn (X.Z.); lzuo@mail.neu.edu.cn (L.Z.)

**Keywords:** shape memory alloy, martensitic transformations temperature, first-principles, machine learning, empirical formula

## Abstract

Shape memory alloys (SMAs) derive their unique functional properties from martensitic transformations, with the martensitic transformation temperature (*T*_M_) serving as a key design parameter. However, existing empirical rules, such as the valence electron concentration (VEC) and lattice volume (V) criteria, are typically restricted to specific alloy families and lack general applicability. In this work, we used a data-driven methodology to find a generalizable empirical formula for *T*_M_ in SMAs by combining high-throughput first-principles calculations, feature engineering, and symbol regression techniques. Key factors influencing *T*_M_ were first identified and a predictive machine learning model was subsequently trained based on these features. Furthermore, an empirical formula of *T*_M_ = $82(\bar{\rho }\cdot \bar{MP})-700$ was derived, where $\bar{\rho }$ and $\bar{MP}$ represent the weight-average value of density and melting point, respectively. The empirical formula exhibits strong generalizability across a wide range of SMAs, such as NiMn-based, NiTi-based, TiPt-based, and AuCd-based SMAs, etc., offering practical guidance for the compositional design and optimization of shape memory alloys.

## 1. Introduction

Shape memory alloys (SMAs) are a class of functional materials renowned for their unique phase transformation behaviors, including the shape memory effect [1], superelasticity [2], and elastocaloric effect [3]. These functionalities originate from martensitic transformation. Among various properties, the martensitic transformation temperature (*T*_M_) is a critical parameter in SMA design, as it directly determines the operational temperature window of the material. For example, Ni_2_MnGa, one of the most extensively studied shape memory alloys, exhibits a *T*_M_ around 220 K, thereby restricting its application in ambient or high-temperature environments such as actuators or sensors. In contrast, cryogenic applications like solid-state cooling require the *T*_M_ to be substantially below room temperature. Therefore, precise control and reliable prediction of the *T*_M_ are essential for tailoring the functional properties of SMAs for diverse applications.

Several strategies have been developed to tune *T*_M_, including modifications of chemical composition, microstructure, atomic ordering, and vacancy concentration [4,5,6,7], with compositional tuning emerging as the most effective approach. Empirical rules, such as the valence electron concentration (VEC) rule [3,8,9,10] where the *T*_M_ generally increases with higher VEC, and the lattice volume rule [11] where a reduced austenite lattice volume tends to raise the *T*_M_, have been widely employed. Based on these principles, empirical models like *T*_M_ = 705(VEC) − 5067 for Ni-Mn-Ga alloys have been proposed [12]. Other composition-related empirical formulas include *T*_M_ = 1000 × (Ni% − 50) for Ni-Ti with 50~60 at.% [13,14], and for Ti-Nb where each 1 at.% increase in Nb content decreases the *T*_M_ by approximately 43 K [15,16]. However, these models often suffer from limited generalizability across different alloy systems due to their strong dependence on specific material families. Therefore, identifying universal key factors that govern *T*_M_ across various shape memory alloys (SMAs) remains a critical challenge. Recently, machine learning (ML) has emerged as a powerful technique to model complex, nonlinear relationships and predict material properties. Although previous studies have applied ML to forecast *T*_M_ in SMAs, they often rely on computationally expensive features derived from density functional theory (DFT) calculations or experimental inputs [17], or are restricted to specific alloy families [18,19]. Therefore, developing fast and generalizable models to predict *T*_M_ based on readily available information holds significant theoretical and practical value.

To address this challenge, we introduced a big data-driven machine learning framework based on elemental and simple substance properties—a state-of-the-art approach for resolving multifactorial materials design problems [18,20,21,22]. In this work, high-throughput ab initio calculations were employed alongside extensive data collection from the literature to expand the training dataset. Subsequently, a five-step descriptor screening process was implemented to identify the key factors governing *T*_M_. Based on these critical features, a highly accurate random forest machine learning model was developed to predict *T*_M_. Furthermore, a simple yet robust empirical relationship between *T*_M_ and the identified parameters was discovered, exhibiting exceptional generalization performance across a wide range of SMAs.

## 2. Methods

A random forest (RF) machine learning model, as implemented in the Scikit-Learn software package (version 1.3.2) [23] was employed in this study. The number of decision trees was set to 50. During model construction, the bootstrap sampling strategy [24] was adopted, and the maximum tree depth was left unconstrained to allow for full data-driven learning. To mitigate the risk of overfitting, 10-fold cross-validation was performed [25]. Model performance was evaluated using the mean absolute error (MAE), root mean square error (RMSE) and mean square error (MSE). To identify the dominant factors influencing *T*_M_, a comprehensive feature selection strategy was employed, involving the variance screening, the Pearson correlation screening [26], the univariate screening [27,28], the recursive elimination screening [29], and the exhaustive screening [21]. Detailed descriptions of these methods can be found in Ref. [22].

High-throughput ab initio calculations were conducted using spin-polarized density functional theory within the framework of the projector augmented-wave (PAW) method [30], as implemented in the Vienna Ab initio Simulation Package (VASP, version 6.4.2) [31]. The exchange-correlation energy was treated using the Perdew-Burke-Enzerh (PBE) functional within the generalized gradient approximation (GGA) [32,33]. The plane-wave kinetic energy cutoff, total energy convergence criterion, and force convergence criterion were set to 600 eV, 10^−5^ eV, and 10^−4^ eV/Å, respectively. A 16-atom supercell was adopted for all calculations. The first Brillouin zone was sampled using a Monkhorst-Pack [34] k-point grid of 13 × 13 × 13. 

## 3. Results and Discussion

### 3.1. Dataset

Based on the literature [17,18,35], a dataset comprising 82 experimental data points of the *T*_M_ in various shape memory alloys was collected. These systems include NiMn-based, NiTi-based, AgCd-based, AuCd-based, PdTi-based, PtTi-based, CoNi-based, TiNb-based, TiTa-based, ZnAuCu-based, TiAu-based, and MgSc-based alloys, effectively covering nearly all known shape memory alloy families to date (details in Appendix A Table A1).

**Figure 1:**
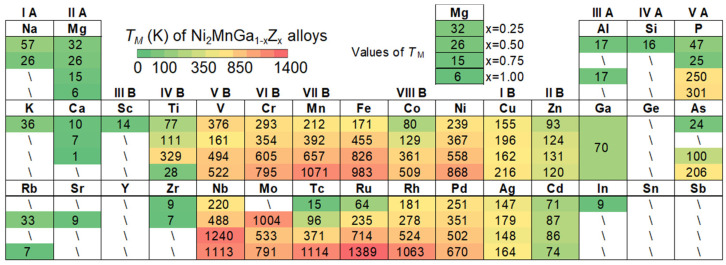
*T*_M_ of Ni_2_MnGa_1−x_Z_x_ alloys (x = 0.25, 0.5, 0.75 and 1) from high-throughput ab initio calculations.

To further expand the dataset, high-throughput ab initio calculations were performed on 109 kinds of L2_1_ structured Ni_2_MnGa_1−x_Z_x_ (x = 0.25, 0.5, 0.75 and 1) alloys, where Z represents various common elements from the periodic table, as illustrated in Figure 1. The Bain distortion model was employed to compute the energy difference (Δ*E*) between the martensitic and austenitic phases (see calculation details in Ref. [22]). The *T*_M_ was then estimated using the relation $T_{M}=\frac{∆E}{nk_{B}}$ [36,37] where *n* is the number of atoms in the supercell, and *k*_B_ is the Boltzmann constant which connects the macroscopic temperature and microscopic energy parameters. Figure 1 displays the calculated martensitic transformation temperatures (*T*_M_) for Ni_2_MnGa_1−x_Z_x_ alloys, where Z spans 35 different substituting elements and x takes values of 0.25, 0.5, 0.75, and 1.00. For each element, corresponding *T*_M_ values are shown beneath the elemental symbol. A backslash symbol (“\”) indicates compositions where the austenitic phase is energetically more stable than the martensitic phase even at 0 K, corresponding to a negative Δ*E*. In these cases, martensitic transformation does not occur, and the alloy remains in a stable austenitic state.

**Figure 2:**
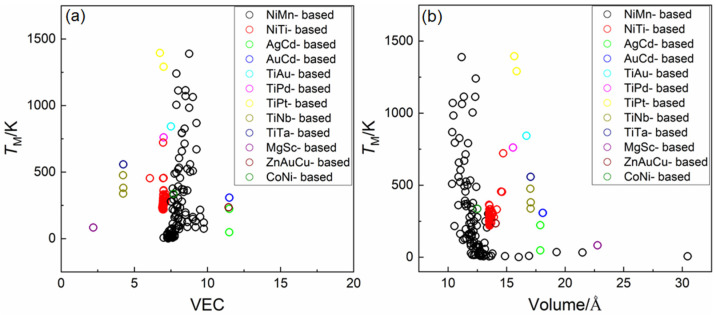
The relationship between the martensitic transformation temperature *T*_M_ of alloys in the dataset and known empirical rules (**a**) VEC rule and (**b**) Volume rule. Neither VEC nor V exhibited a strong or reliable correlation with *T*_M_ when considering the full dataset.

To assess the applicability of known empirical rules across this heterogeneous dataset, the relationships between *T*_M_ and two classic descriptors were examined: Figure 2a,b shows the correlations of the *T*_M_ with the valence electron concentration (VEC) and lattice volume (V), respectively. Although prior studies have demonstrated that an increase in VEC or a decrease in lattice volume correlates tends to elevate the *T*_M_ within specific alloy families [3,8,9,10,11,12] these trends do not consistently extend across a broader and more diverse dataset. In our analysis, neither VEC nor V exhibited a strong or reliable correlation with *T*_M_ when considering the full dataset, thereby underscoring the limited generalizability of these traditional empirical rules. In addition, the transformation temperatures span a wide range— from approximately 100 K to 1300 K—reflecting the intrinsic diversity of thermal responses among different SMA compositions. This rich and representative dataset thus provides a robust foundation for the development of data-driven predictive models and enables cross-system generalization.

### 3.2. Feature Engineering

To represent the different alloys in the training set, a total of 64 constituent element-associated parameters were initially considered, given that the structural phase transition as well as the physical and chemical properties of compounds are predominantly determined by their constituent elements [38,39,40,41]. Specifically, the weight-average value and standard deviation of 31 types of element or elemental-substance-associated parameters were calculated for each alloy [39,40,42], as summarized in Table 1.

In addition to these elemental descriptors, two phase stability-related parameters, i.e., mixing entropy $∆S_{mix}$ and atom radium difference δ [20], were included. Considering that shape memory alloys generally possess mixed metallic, covalent, and ionic chemical bonds [43,44], three different atom radius definitions, including atomic radius, covalent radius, and ionic radius, were employed to estimate δ. In total, 64 descriptors were utilized to featurize the alloys within the training dataset.

### 3.3. Screening of Key Features

**Figure 3:**
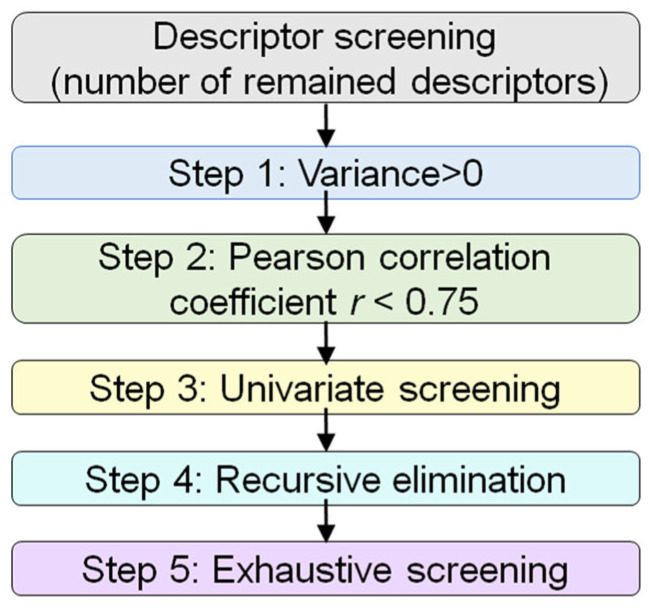
Workflow of adopted five-step descriptor screening.

To eliminate the redundant or insignificant descriptors and to identify the key materials parameters governing *T*_M_, a successive five-step screening procedure was employed, as illustrated in Figure 3. This process combined a series of complementary descriptor selection methods applied sequentially: variance screening (Step 1) [45], Pearson correlation screening (Step 2) [26], univariate feature selection (Step 3) [27,28], recursive feature elimination (Step 4) [29], and exhaustive screening (Step 5) (more details in Appendix B and Ref. [21]). The detailed description of these selection methods can be found in Ref. [22].

First, redundancy among descriptors was eliminated using the variance method (Step 1) [45], by removing descriptors that exhibited identical values across all data points in the training set. Second, strongly correlated descriptors were identified through Pearson correlation screening (Step 2) [26]. The Pearson correlation coefficient (*r*) between every pair of descriptors was computed, and for each pair with |*r|* > 0.75, one descriptor was removed to reduce redundancy. For instance, the atomic number (Z) and atomic weight (AW) showed a perfect correlation (*r* = 1); thus, one of the two, such as the atomic number in this case, was eliminated. Following this screening process, 27 descriptors were retained for further analysis.

**Figure 4:**
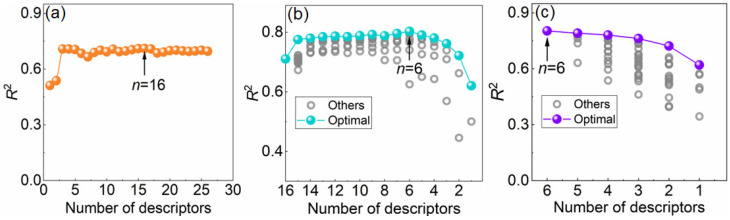
Machine learning screening of key parameters affecting martensitic transformation temperature. (**a**) Univariate screening; (**b**) Recursive elimination; and (**c**) Exhaustive screening.

Figure 4a–c presents the results of feature selection using univariate filtering, recursive feature elimination (RFE), and exhaustive search, respectively. Following these three successive screening steps, the number of features was reduced from the initial 66 to 16, and finally to 6. This systematic feature selection process effectively narrowed the original set of candidate descriptors down to six key features, which are summarized in Table 2.

The final six descriptors identified as critical for determining the martensitic transformation temperature (*T*_M_) are: the weight-average value of ionization energy ($\bar{IE}$), weight-average value of heat capacity ($\bar{HC}$), weight-average value of shear modulus ($\bar{G}$), and standard deviation of atomic radius (σ_AR_). These descriptors were subsequently used to train the machine learning models, as they represent the dominant factors influencing *T*_M_.

Using the six final selected features summarized in Table 2, a random forest (RF) regression model was retrained to predict the martensitic transformation temperature (*T*_M_) of the alloys. The model’s performance was evaluated through a ten-fold cross-validation procedure to ensure its generalization ability and predictive accuracy. The resulting coefficient of determination (*R*^2^) reached 0.82, demonstrating that the model exhibits excellent predictive performance.

**Figure 5:**
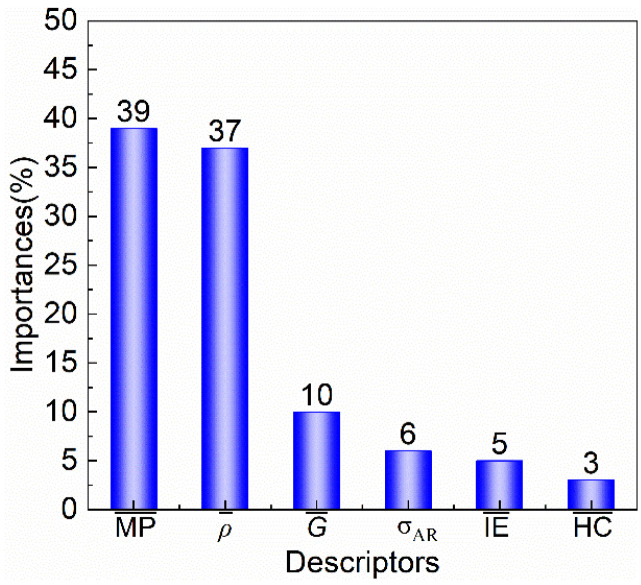
The result of 10-cross validation and the importance of descriptors in machine learning models.

Figure 5 presents the feature importance ranking obtained from the random forest model. By assessing the relative contribution of each feature to the model’s prediction capability, the following order of importance was determined: $\bar{MP}$(39) > $\bar{\rho }$ (37) > $\bar{G}$ (10) > σ_AR_ (6) > $\bar{IE}$ (5) > $\bar{HC}$(3). These results highlight the dominant roles of melting point and density in governing the *T*_M_ of shape memory alloys.

### 3.4. Linear Relationship Between $\bar{\rho }\cdot \bar{MP}$ and T_M_

To further investigate the influence of the six key features identified by machine learning on the martensitic transformation temperature (*T*_M_) of shape memory alloys (SMAs), detailed visual analyses were conducted.

**Figure 6:**
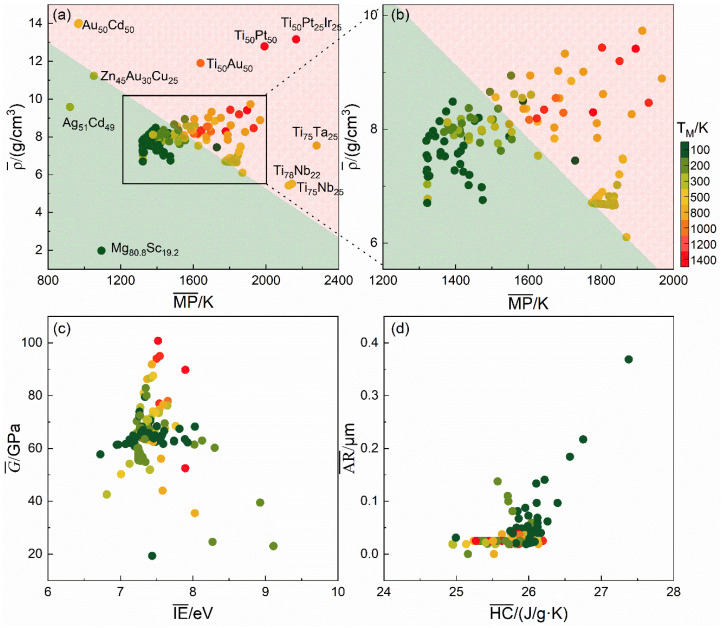
The visualization of the relationship between the target *T*_M_ and the screened key descriptors. (**a**) $\bar{MP}$ and $\bar{\rho }$; (**b**) Enlarged view of the black rectangular region in panel (**a**); (**c**) $\bar{G}$ and $\bar{IE}$; (**d**) σ_AR_ and $\bar{HC}$.

Figure 6a illustrates the relationship between the weight-average value of density ($\bar{\rho }$), the weight-average value of melting point ($\bar{MP}$), and *T*_M_. As shown, the feature space defined by $\bar{\rho }$ and $\bar{MP}$ can be effectively divided into two distinct regions corresponding to different *T*_M_ ranges. The green region in the lower left corner is characterized by low values of both $\bar{MP}$ and $\bar{\rho }$; this region corresponds to relatively low martensitic transformation temperatures (*T*_M_ < 300 K). The red region in the upper right corner is marked by higher values of both $\bar{MP}$ and $\bar{\rho }$; this region corresponds to higher transformation temperatures (*T*_M_ > 300 K). This separation suggests that $\bar{MP}$ and $\bar{\rho }$ act as discriminative features that jointly determine *T*_M_ in SMAs. Figure 6b provides an enlarged view of the black-boxed region from Figure 6a, further confirming the observed trend: higher values of the weight-average of melting point ($\bar{MP}$) and weight-average value of density ($\bar{\rho }$) correspond to higher martensitic transformation temperatures (*T*_M_). This observation reinforces the conclusion that increases in $\bar{MP}$ and $\bar{\rho }$ are closely associated with elevated *T*_M_ values. Figure 6c illustrates the relationships between the weight-average value of shear modulus ($\bar{G}$), the weight-average value of ionization energy ($\bar{IE}$), and *T*_M_. The results reveal weak correlations between these features and *T*_M_, suggesting that $\bar{G}$ and $\bar{IE}$ have a minimal influence on the martensitic transformation temperature. Similarly, Figure 6d depicts the relationships between the standard deviation of atomic radius (σ_AR_), the weight-average value of heat capacity ($\bar{HC}$), and *T*_M_. Again, no clear trends are observed, indicating that these descriptors also exert relatively minor influence on *T*_M_. In sum, the weight-average value of melting point ($\bar{MP}$) and weight-average value of density ($\bar{\rho }$) are identified as the dominant factors affecting the martensitic transformation temperature. Consequently, these two features were selected for the construction and analysis of an empirical predictive formula.

Using $\bar{MP}$ and $\bar{\rho }$ as the two key features, a symbolic regression approach was employed to develop an empirical formula for predicting the martensitic transformation temperature (*T*_M_) of SMAs. Symbolic regression [46] is a data-driven technique for discovering functional relationships, aiming to autonomously derive optimal analytical expressions within a predefined mathematical operator space. In this study, symbolic regression was implemented using a genetic algorithm [46]. The mathematical operator space consisted of basic arithmetic operations, including addition, multiplication, subtraction, and division. The input features were $\bar{MP}$ and $\bar{\rho }$, and the target variable was *T*_M_. The fitness of each candidate formula was evaluated based on the correlation coefficient (*r*) between the predicted and actual *T*_M_ values.

After 500 generations of iterative optimization, the final empirical formula was identified:

$$T_{M}=82(\bar{\rho }\cdot \bar{MP})-700$$

where $\bar{\rho }$ and $\bar{MP}$ represent weight-average values of density and melting point, respectively, and *T*_M_ is the martensite transition temperature of SMAs. This result highlights that *T*_M_ scales approximately linearly with the combined descriptor $\bar{\rho }\cdot \bar{MP}$.

**Figure 7:**
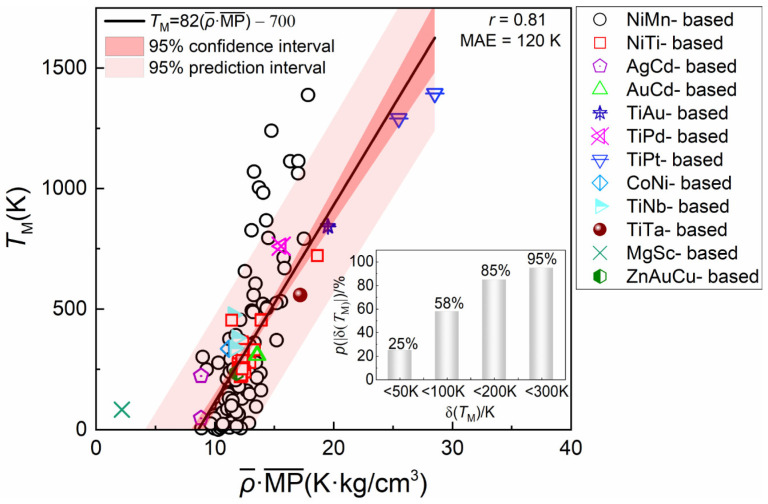
The visualization of the relationship between the *T*_M_ and $\bar{\rho }\cdot \bar{MP}$ of the dataset. The black line represents the fitted empirical formula *T*_M_ = $82(\bar{\rho }\cdot \bar{MP})-700$. The red and pink shaded region correspond to the 95% confidence interval and 95% prediction interval, respectively. The inset plot shows the distribution of prediction errors.

Figure 7 illustrates the relationship between $\bar{\rho }\cdot \bar{MP}$ and *T*_M_ across the entire dataset. A strong linear correlation is observed (*r* = 0.81 and MAE = 120 K), confirming that the empirical relationship generated via symbolic regression accurately captures the dependence of *T*_M_ on the key features. Additionally, the distribution of the absolute error δ(*T*_M_) is shown in the inset of Figure 7. Notably, for the majority of datapoints (58%), δ(*T*_M_) is less than 100 K. To further evaluate the model’s performance, both the 95% confidence interval and the 95% prediction interval were constructed in Figure 7. Most data points fall within the prediction intervals, are closely aligned with the confidence intervals, and are distributed symmetrically around the regression line. These results indicate that the model provides an accurate and reliable estimation of the overall trend in *T*_M_ across different compositions.

This simple formula offers an effective method to predict *T*_M_ across a diverse range of alloy families, including NiTi-based, TiPd-based, NiMn-based, TiAu-based, MgSc-based, AgCd-based, AuCd-based, PdTi-based, PtTi-based, CoNi-based, TiNb-based, TiTa-based, and ZnAuCu-based SMAs. This stands in contrast to traditional empirical rules such as the valence electron concentration (VEC) and lattice volume (V) rules, or other composition-related rules, which are typically applicable only to specific alloy families (see Figure 2 and Refs. [3,8,9,10,11,12]). While previous studies have used ML to forecast *T*_M_ in SMAs, many faced similar limitations to traditional empirical approaches: (1) some models are restricted to specific alloy systems [18,19], and (2) models capable of predicting across multiple systems often rely on computationally expensive features derived from density functional theory (DFT) calculations and require complex frameworks such as artificial neural networks [17]. These DFT-based models can take hours or even days to process a single data point, and their computational cost increases rapidly with prediction set size. In contrast, the empirical formula proposed in this work enables near real-time predictions of *T*_M_ for hundreds or even thousands of alloy compositions, as it relies solely on simple substance properties that are readily available. The key advantage of our formula lies in its ability to quickly and efficiently predict the martensitic transformation temperature across multiple SMA systems.

## 4. Conclusions

In this study, a systematic data-driven approach was developed to derive an interpretable and generalizable empirical formula for predicting the martensitic transformation temperature (*T*_M_) of shape memory alloys (SMAs). By integrating high-throughput first-principles calculations, extensive feature selection techniques, and symbolic regression methods, we identified the key physical factors that govern *T*_M_ behavior. The final empirical model, expressed as *T*_M_ = $82(\bar{\rho }\cdot \bar{MP})-700$, highlights the combined influence of material density and melting point on martensitic transformation characteristics. The proposed formula demonstrates strong predictive capability across diverse classes of SMAs, such as NiMn-based, NiTi-based, TiPt-based, and AuCd-based SMAs, etc. The reliability of the model was quantitatively assessed by evaluating performance metrics and the associated confidence/prediction intervals were reported to ensure accuracy of the model. Compared to previous empirical rules that were often limited to narrow alloy families, the present work offers a unified and practically applicable tool for SMA composition design, offering significant potential for high-throughput material design and optimization.

## Figures and Tables

**Table 1:** Adopted elemental, simple substance, and phase stability-associated features.

Feature Category	Feature Description	Abbreviation
Elemental properties	Atomic number	Z
	Periodic table column	C
	Atomic weight	AW
	Mendeleev number	MN
	Periodic table row	PR
	Atomic radius	AR
	Number of *s* valence electrons	Ns
	Number of *p* valence electrons	Np
	Number of *d* valence electrons	Nd
	Number of *f* valence electrons	Nf
	Number of total valence electrons	Nt
	Number of unfilled *s* states	Us
	Number of unfilled *p* states	Up
	Number of unfilled *d* states	Ud
	Number of unfilled *f* states	Uf
	Number of total unfilled states	Ut
Simple substance properties	Melting point	MP
	Boiling point	BP
	Heat capacity	HC
	Heat fusion	HF
	Pauling Electronegativity	EN
	Covalent radius	CR
	Ionic radius	IR
	Density	$\bar{\rho }$
	Magnetic moment	M
	Volume	V
	Band gap	Gap
	First ionization energy	E
	Space group number	SG
	Bulk modulus	B
	Shear modulus	G
Phase stability properties	Mixing entropy	$∆S_{mix}$
	Atomic size difference	δ

**Table 2:** The selected descriptors for the univariate screening, recursive elimination, and exhaustive screening methods for the dataset.

			Descriptors (Abbr.)	Descriptors (Abbr.)
Univariate screening			Standard deviation of melting point (σ_MP_)	Weight-average value of *p* valence electron ($\bar{VE}$*_p_*)
			Weight-average value of valence electron ($\bar{VE}$)	Weight-average value of *s* valence electron ($\bar{VE}$*_s_*)
			Weight-average value magnetic moment ($\bar{Mag}$)	Weight-average value of bulk modulus ($\bar{G}$)
			Standard deviation of Mendeleev number (σ_MN_)	Standard deviation of heat fusion (σ_HF_)
			Standard deviation of valence electron (VE_sd_)	Standard deviation of *s* valence electron (σ_VEs_)
	Recursive elimination screening	Exhaustive screening	Weight-average value of melting point ($\bar{MP}$)	Standard deviation of atomic radius (σ_AR_)
			Weight-average value of ionization energy ($\bar{IE}$)	Weight-average value of shear modulus ($\bar{G}$)
			Weight-average value of heat capacity ($\bar{HC}$)	Weight-average value of density ($\bar{\rho }$)

**Figure A1:**
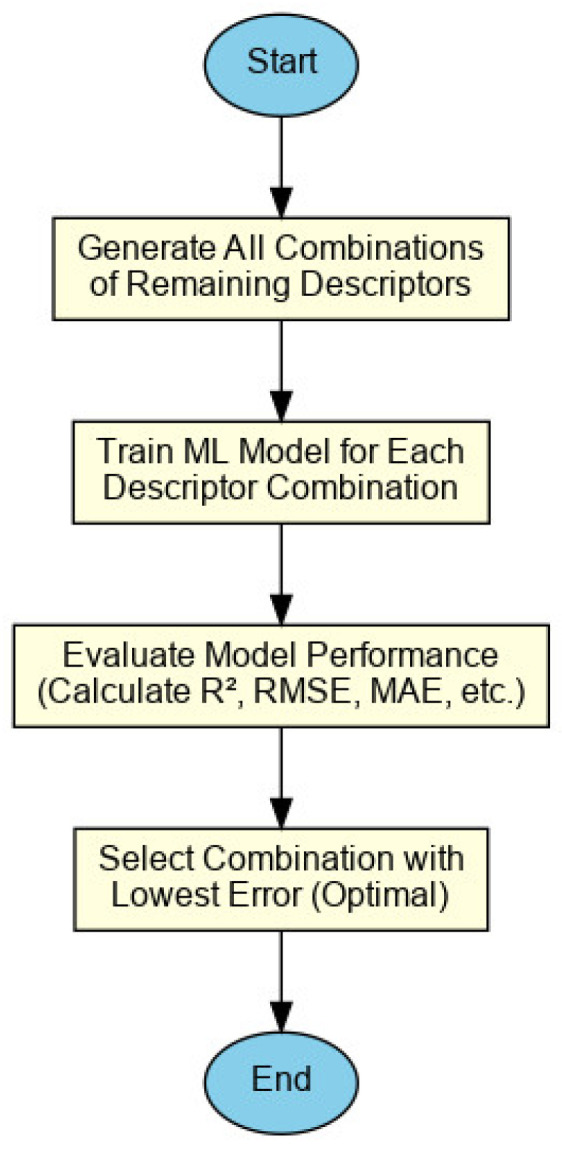
Workflow of exhaustive screening.

## Data Availability

The original contributions presented in this study are included in the article. Further inquiries can be directed to the corresponding author.

## Appendix A

**Table A1:** Chemical compositions and T_M_ values of alloys from experimental (Exp.) in the literature.

Compositions	*T*_M_ (K)	Ref.	Compositions	*T*_M_ (K)	Ref.
Ti_500_Ni_452_Cu_10_Fe_38_	248	[14]	Ti_500_Ni_468_Cu_9_Fe_20_Pd_3_	290	[14]
Ti_500_Ni_444_Cu_20_Fe_36_	239	[14]	Ti_500_Ni_442_Cu_19_Fe_38_Pd_1_	243	[14]
Ti_500_Ni_428_Cu_40_Fe_32_	231	[14]	Ti_500_Ni_467_Cu_8_Fe_23_Pd_2_	282	[14]
Ti_500_Ni_436_Cu_30_Fe_34_	242	[14]	Ti_500_Ni_442_Cu_19_Fe_39_	242	[14]
Ti_500_Ni_460_Cu_0_Fe_40_	234	[14]	Ti_500_Ni_481_Cu_2_Fe_15_Pd_2_	302	[14]
Ti_500_Ni_445_Cu_15_Fe_30_Pd_10_	223	[14]	Ti_500_Ni_445_Cu_16_Fe_37_Pd_2_	244	[14]
Ti_500_Ni_340_Cu_130_Pd_30_	299	[14]	Ti_500_Ni_482_Cu_6_Fe_9_Pd_3_	320	[14]
Ti_500_Ni_340_Cu_100_Pd_60_	303	[14]	Ti_500_Ni_465_Cu_11_Fe_22_Pd_2_	284	[14]
Ti_500_Ni_340_Cu_120_Pd_40_	311	[14]	Ti_500_Ni_483_Fe_16_Pd_1_	302	[14]
Ti_500_Ni_420_Cu_50_Fe_30_	226	[14]	Ti_500_Ni_490_Fe_2_Pd_8_	358	[14]
Ti_500_Ni_350_Cu_120_Pd_30_	321	[14]	Ti_500_Ni_486_Fe_9_Pd_5_	332	[14]
Ti_500_Ni_400_Pd_100_	279	[14]	Ti_500_Ni_435_Cu_20_Fe_45_	226	[14]
Ti_500_Ni_340_Cu_140_Pd_20_	319	[14]	Ti_500_Ni_250_Pd_250_	456	[14]
Ti_500_Ni_340_Cu_160_	333	[14]	Ag_51_Cd_49_	48	[13]
Ti_500_Ni_440_Cu_10_Fe_10_Pd_40_	239	[14]	Au_505_Cd_495_	308	[13]
Ti_500_Ni_340_Pd_160_	331	[14]	Au_50_Ti_50_	843	[13]
Ti_500_Ni_364_Cu_120_Fe_16_	286	[14]	Pd_50_Ti_50_	761	[13]
Ti_500_Ni_412_Cu_60_Fe_28_	220	[14]	Pt_50_Ti_50_	1291	[13]
Ti_500_Ni_380_Cu_100_Fe_20_	274	[14]	Ni_50_Ti_50_	252	[13]
Ti_500_Ni_348_Cu_140_Fe_12_	271	[14]	Co_50_Ni_24_Ga_26_	336	[13]
Ti_500_Ni_396_Cu_80_Fe_24_	258	[14]	Ti_505_Ni_245_Pd_250_	454	[13]
Ti_500_Ni_500_	364	[14]	Ti_505_Ni_245_Pt_250_	721	[13]
Ti_500_Ni_440_Cu_20_Fe_40_	220	[14]	Ti_50_Pt_25_Ir_25_	1395	[13]
Ti_500_Ni_445_Cu_21_Fe_34_	248	[14]	Ni_50_Mn_25_Ga_25_	278	[13]
Ti_500_Ni_440_Cu_23_Fe_36_Pd_1_	241	[14]	Ti_75_Nb_22_	476	[13]
Ti_500_Ni_404_Cu_46_Fe_10_Pd_40_	270	[14]	Ti_75_Nb_25_	381	[13]
Ti_500_Ni_457_Fe_43_	231	[14]	Ti_75_Ta_25_	558	[13]
Ti_500_Ni_458_Fe_42_	234	[14]	Mg_81_Sc_19_	83	[13]
Ti_500_Ni_428_Cu_36_Fe_28_Pd_8_	234	[14]	Ag_51_Cd_49_	223	[30]
Ti_500_Ni_439_Cu_21_Fe_40_	233	[14]	Au_50_Cd_50_	308	[30]
Ti_500_Ni_445_Cu_17_Fe_37_Pd_1_	245	[14]	Ti_50_Au_50_	843	[30]
Ti_500_Ni_457_Cu_12_Fe_30_Pd_1_	264	[14]	Zn_45_Au_30_Cu_25_	235	[30]
Ti_500_Ni_440_Cu_23_Fe_37_	234	[14]	Co_50_Ni_24_Ga_26_	336	[30]
Ti_500_Ni_451_Cu_14_Fe_35_	248	[14]	Ti_76_Nb_24_	338	[30]
Ti_500_Ni_446_Cu_19_Fe_34_Pd_1_	250	[14]	Ni_50_Mn_25_Ga_25_	278	[30]
Ti_500_Ni_438_Cu_26_Fe_36_	239	[14]	Ti_50_Ni_50_	252	[30]
Ti_500_Ni_439_Cu_15_Fe_46_	228	[14]	Ti_50_Pd_50_	761	[30]
Ti_500_Ni_445_Cu_19_Fe_34_Pd_2_	243	[14]	Ti_50_Pt_50_	1291	[30]
Ti_500_Ni_460_Cu_11_Fe_28_Pd_1_	261	[14]	Ti_50.5_Ni_24.5_Pd_25_	454	[30]
Ti_500_Ni_438_Cu_20_Fe_41_Pd_1_	231	[14]	Ti_50.5_Ni_24.5_Pt_25_	721	[30]
Ti_500_Ni_439_Cu_20_Fe_40_Pd_1_	232	[14]	Ti_50_Pt_25_Ir_25_	1395	[30]

## Appendix B

The exhaustive method was used to identify the best combination of descriptors. Every possible combination of the remaining descriptors after the recursive elimination was used to train the ML model. The combination of descriptors in which the ML model possesses the lowest error was selected as the critical factors that determine the *T*_M_ of SMA. The detailed flow is as follows (Figure A1):

(i) Generate All Combinations of Remaining Descriptors: After eliminating irrelevant features, every possible combination of the remaining descriptors is generated for testing.
(ii) Train ML Model: Each combination of descriptors is used to train a ML model, ensuring a diverse exploration of feature subsets.
(iii) Evaluate Model Performance: The performance of each model is evaluated using standard error metrics like *R*^2^, RMSE, and MSE to assess its accuracy.
(iv) Select Optimal Combination: The combination of descriptors that results in the lowest error is chosen as the most significant contributors to the *T*_M_ prediction.
